# Seeing clearly without your lenses: digital holographic microscopy analysis of blood culture Gram stains

**DOI:** 10.1128/jcm.00467-26

**Published:** 2026-08-28

**Authors:** Mark D. Gonzalez

**Affiliations:** 1 Department of Pathology, Children’s Healthcare of Atlanta, Atlanta, Georgia, USA; Endeavor Health, Evanston, Illinois, USA

## Abstract

While automation and digital imaging technologies are now available for setup and workup of microbiology cultures, specimen Gram stain reading is still manually performed and requires experience to provide accurate results. In the article by K. P. Smith, E. Boulanger, M. Elkan, J. Fink, et al. (J Clin Microbiol 64:e01627-25, 2026, https://doi.org/10.1128/jcm.01627-25), the authors use a non-optical-based methodology, digital holographic microscopy, in combination with artificial intelligence to automate and provide interpretations and digital images of Gram stains from positive blood culture broths. While this differs from previous optical-based methods for Gram stain slide scanning, it demonstrated comparable performance. Gram stain slide staining and assistance by artificial intelligence for slide interpretation are a tantalizing future tool for clinical microbiology laboratories.

## COMMENTARY

The application of artificial intelligence to clinical microbiology offers an opportunity to help medical laboratory scientists with the evaluation of culture plates, Gram stain reading, parasite examination, and with other areas (1). In the case of Gram stain and parasite examination, it first requires the capturing of slide images for artificial intelligence analysis. Mathison et al. utilized a 40× objective lens for scanning trichrome-stained stool samples that were then analyzed by a highly trained artificial intelligence model, which demonstrated a >98% positive and negative predictive values for protozoal parasite identification (2). In a study by Walter et al., 1,555 positive blood culture Gram stain slides were scanned using a 40× oil objective lens, and, with artificial intelligence, analysis showed a positive percent agreement of 95.8% for gram-positive cocci in clusters, 87.6% for gram-positive cocci in pairs and chains, and 97.4% for rod-shaped bacteria (3). In a separate study, Smith et al. scanned 189 positive blood culture Gram stain slides with a dry 40× objective lens for automated analysis of Gram stain interpretation by an artificial intelligence model (4). They found correct Gram stain identifications of 98.1, 97.2, and 75% for gram-negative bacilli, gram-positive cocci in clusters, and gram-positive cocci in chains, respectively (4). In all of these above examples, image acquisition was performed with optical lenses.

In the article entitled “Digital holographic microscopy: use of artificial intelligence to enable rapid blood culture Gram stain digitization and interpretation,” the authors used a non-optical lens method, digital holographic microscopy (DHM), in combination with artificial intelligence for Gram stain evaluation of positive blood culture slides (5). There are numerous applications of DHM, including label-free analysis of urine samples as a screening method compared to urine cultures (6) or the study of magnetotaxis dynamics of magnetotactic bacteria (7). DHM uses multispectral coherent light to scan slides, and then the diffracted light from single wavelengths of light are digitally captured as a collection of monochromatic holograms, and these individual holograms are assembled for final image construction.

For this study, Smith et al. manually prepared Gram stain slides in duplicate from positive BD BacTec Peds Plus/F pediatric bottles (Becton Dickinson, Sparks, MD), heat-fixed, and manually Gram stained (5). One of the slides was read and reported for clinical purposes, while the other slide was reserved for this study. After review of blood culture results, only monomicrobial blood cultures with gram-positive cocci in clusters, gram-positive cocci in chains, and gram-negative bacilli were included for further evaluation.

Gram-stained slides were scanned for DHM using the MVS 100 scanner. Since Gram stain slides can be non-uniform in the thickness and distribution of the specimen, an automated quality assessment was done with training Gram-stained slides to identify suitable slide areas for additional evaluation, and computational analysis was performed to focus on the proper vertical specimen plane. Next, the individual holograms meeting the quality assessment and focus criteria were applied to a neural network for image reconstruction. All the above scanning and analysis on the MVS 100 scanner take 4 min per slide.

The authors applied this DHM method to 77 blood culture Gram stain slides with an automated interpretation, with 65 slides considered acceptable for further evaluation. Of the 15 slides with gram-negative bacilli, 14 (93.3%) were correctly identified, with one misclassified as gram-positive cocci in clusters. Of the 42 slides with gram-positive cocci in clusters, 40 (95.2%) were correctly identified, with one misidentified as gram-negative bacilli and the other as gram-positive cocci in chains. Finally, of the eight slides with gram-positive cocci in chains, six (75%) were correctly identified, while one was misidentified as gram-negative bacilli and the other as gram-positive cocci in clusters.

To examine how medical laboratory scientists would interpret DHM Gram stains, representative images from 16 slides (gram-negative bacilli, *n* = 6; gram-positive cocci in clusters, *n* = 4; and gram-positive cocci in chains, *n* = 6) were evaluated by six medical laboratory scientists in an interactive portal without an automated identification provided. Gram stain results could be reported as one of the three listed morphologies or as indeterminate if the medical laboratory scientist could not determine the Gram stain reaction and/or the organism morphology. For the gram-negative bacilli, 94.4% was correctly identified, with two results classified as indeterminate. For gram-positive cocci in clusters, 87.5% was correctly identified, with three results classified as indeterminate. For the gram-positive cocci in chains, 88.9% was correctly identified with three classified as indeterminate and one misidentified as gram-negative bacilli since the laboratory scientist thought it was under-decolorized gram-negative coccobacilli. When focusing on the 88 non-indeterminate results, 87 (98.9%) were correctly classified. Of the eight indeterminate classified results, three slides were reported as such by more than one laboratory scientist, and the authors noted that indeterminate slides tended to have a lower amount of organism.

This initial promising report of DHM for Gram stain interpretation utilized positive blood culture broth specimens, which tend to be monomicrobial, have high organism burden, and are a relatively simple specimen type compared to direct clinical samples (e.g., respiratory or cerebrospinal fluid). Also, the authors focused on three common blood culture Gram stain morphologies for this proof-of-concept study. Thus, the next steps would be to evaluate a larger set of these three morphologies along with others that are encountered in monomicrobial blood cultures, such as gram-negative diplococci, gram-negative coccobacilli, yeast, and gram-positive bacilli, along with polymicrobial blood cultures. Additionally, it would be beneficial to confirm these results with other blood culture media types and brands, be replicated by other laboratories, and use methanol-fixed slides, which is the recommended fixation method for blood culture and specimen slide preparation (8). Given that 8.3% of the medical laboratory scientist results were indeterminate, it would be important to determine if automated Gram stain slide instruments would help reduce this rate. Finally, applying DHM to direct patient specimens, which are more heterogeneous and can have lower organism burden than positive blood culture broth, is needed.

The other reports of artificial intelligence reading of blood culture Gram stains used optical lens for scanning and showed comparable results to those presented with DHM. Whether DHM or optical lens scanning offers superior image data for artificial intelligence analysis of Gram stains remains to be determined, as well as whether one method is superior to other parameters (e.g., number of indeterminate results, incorrectly classified artifacts, etc). Regardless of the slide scanning methodology, it may become more commonplace in clinical microbiology laboratories, as is occurring for anatomic pathology (9). However, compared to the storage of Gram-stained slides in the laboratory, data storage of scanned slides requires laboratories to consider the additional costs and security issues of maintaining this data.

Some potential advantages of artificial intelligence analysis of Gram stains include assisting medical laboratory scientists and directors with infrequently seen organisms (e.g., *Brucella* spp., *Francisella tularensis*) or unusual presentations seen in Gram stains, such as *Strongyloides stercoralis* larvae in a tracheal aspirate (10) or *Plasmodium falciparum* from a positive blood culture (11). Repositories of Gram stain images would help with training new laboratory staff and for clinical colleagues. Additionally, if it is known that specific images are used for Gram stain reporting, those can be retrospectively reviewed when there are corrected reports and discrepancies with the culture results. This could also be done with missed proficiency testing surveys and assist with overall Gram stain quality improvement in the laboratory. Digital Gram stain images would also be useful for assisting laboratory staff that infrequently perform Gram stains, such as laboratory generalists and/or offsite laboratories, with microbiology specialists reviewing them and helping with Gram stain interpretation.

In summary, the article by Smith et al. evaluates a new, non-optical-based methodology for image acquisition of blood culture Gram stains that, in combination with artificial intelligence, provides interpretation assistance. Further work is needed to determine the best method for slide scanning, optical or DHM, however, the artificial intelligence algorithms for analysis will likely improve such that Gram stain reading may no longer be a completely manual process. Clinical microbiology laboratories will need to learn the best ways to incorporate this technology to help improve patient care and assist with laboratory staff workloads.
